# P2P proteomics -- data sharing for enhanced protein identification

**DOI:** 10.1186/1759-4499-4-1

**Published:** 2012-01-31

**Authors:** Marco Schorlemmer, Joaquín Abián, Carles Sierra, David de la Cruz, Lorenzo Bernacchioni, Enric Jaén, Adrian Perreau de Pinninck, Manuel Atencia

**Affiliations:** 1 Artificial Intelligence Research Institute, IIIA-CSIC, Spain; 2 CSIC/UAB Proteomics Laboratory, IIBB-CSIC, IDIBAPS, Spain

## Abstract

**Background:**

In order to tackle the important and challenging problem in proteomics of identifying known and new protein sequences using high-throughput methods, we propose a data-sharing platform that uses fully distributed P2P technologies to share specifications of peer-interaction protocols and service components. By using such a platform, information to be searched is no longer centralised in a few repositories but gathered from experiments in peer proteomics laboratories, which can subsequently be searched by fellow researchers.

**Methods:**

The system distributively runs a data-sharing protocol specified in the Lightweight Communication Calculus underlying the system through which researchers interact via message passing. For this, researchers interact with the system through particular components that link to database querying systems based on BLAST and/or OMSSA and GUI-based visualisation environments. We have tested the proposed platform with data drawn from preexisting MS/MS data reservoirs from the 2006 ABRF (Association of Biomolecular Resource Facilities) test sample, which was extensively tested during the ABRF Proteomics Standards Research Group 2006 worldwide survey. In particular we have taken the data available from a subset of proteomics laboratories of Spain's National Institute for Proteomics, *ProteoRed*, a network for the coordination, integration and development of the Spanish proteomics facilities.

**Results and Discussion:**

We performed queries against nine databases including seven *ProteoRed *proteomics laboratories, the NCBI Swiss-Prot database and the local database of the CSIC/UAB Proteomics Laboratory. A detailed analysis of the results indicated the presence of a protein that was supported by other NCBI matches and highly scored matches in several proteomics labs. The analysis clearly indicated that the protein was a relatively high concentrated contaminant that could be present in the ABRF sample. This fact is evident from the information that could be derived from the proposed P2P proteomics system, however it is not straightforward to arrive to the same conclusion by conventional means as it is difficult to discard organic contamination of samples. The actual presence of this contaminant was only stated after the ABRF study of all the identifications reported by the laboratories.

## Background

Proteomics studies the quantitative changes occurring in a proteome and its application for disease diagnostics and therapy, and drug development. It examines proteins at different levels, including their sequences, structures and functionalities, and it is considered the next step in the study of biological systems, after genomics. It is much more complicated than genomics mostly because the proteome differs from cell to cell and changes constantly through its biochemical interactions with the genome and the environment, while the genome of an organism is rather constant.

Proteins are large linear chains of amino-acids (residues). The sequence of amino-acids in a protein is directly translated from the information encoded in the genome. However, a proteome is more complex than a genome. One organism has radically different protein expression in different parts of its body, different stages of its life cycle and different environmental conditions (e.g., in humans there are about 20,500 identified genes but an estimate of more than 500,000 proteins that are derived from these genes [1]). This is mainly caused by mRNA alternative splicing processes and by the possibility of residues in a protein being chemically altered in post-translational modification (PTM), either as part of the protein maturation processes before the protein takes part in the cell's functionalities, or as part of control mechanisms. The discrepancy implies that protein diversity cannot be fully characterized by gene expression analysis. Thus, proteomics is necessary for a better characterization of cells and tissues, and for manufacturing improved drugs and medicines.

### Protein Identification in Proteomics

One important and challenging task in proteomics is the identification of proteins, that is, the recognition of the sequenced protein if the protein is known, or its discovery if it is unknown. For this, protein sequences are stored in public databases (such as *nrNCBI, UniProt*, or *Genpept*). However, they are mostly produced by the direct translation of gene sequences. This means that neither proteins with post-translation modifications (PTM) nor proteins whose genomes have not been sequenced would find exact matches in such databases.

A key experimental technique for the identification of proteins is mass spectrometry (MS). Mass spectra provide very detailed fingerprints of the proteins contained in a given sample. In the so called shotgun approach, MS is often combined with cutting-edge separation technologies to allow large-scale analysis of proteomes. For this, proteins are extracted from cells and tissues, enzymatically digested, and the resulting peptides (shorter amino-acids chains) separated by multidimensional liquid chromatography techniques. As the peptides are separated, they are on-line injected into the mass spectrometer, where they are ionized, fragmented and these fragments mass-monitored to produce a specific sequence fingerprint.

Identification of the huge amount of spectra produced by current state-of-the-art high-throughput analysis is one of the major tasks for proteomics laboratories. Mainly two popular bioinformatics techniques are involved in this effort. The first one takes advantage of public genome-translated databases (GTDB) that can be accessed through data-mining software (search engines), which directly relates mass spectra with database sequences. Most of these search engines (*Mascot, X! Tandem, SEQUEST, OMSSA*) are available both as stand-alone programs that consult a local copy of a GTDB, or as web-services connected to online GTDBs. The limitations, once again, lie in their capability of identifying missing PTMs or unsequenced genomes. The latter case is addressed applying *de novo *interpretation algorithms that yield a sequence for a given mass spectrum, thus avoiding any database search. But these algorithms cannot become a solution of the problem because of intrinsic technical limitations. Once a protein has been sequenced *de novo*, one can look for similar proteins in a GTDB using a matching algorithm such as BLAST [2] or FASTA [3]; or, alternatively, one can use an algorithm such as OMSSA [4] to match spectra directly to sequences of a GTDB.

Mass spectra identification is usually carried out by mixing and combining these two techniques. However, among other factors, the following issues complicate this task: the number of possible PTMs can multiply the amount of results to be analysed; bad quality and noise in mass spectra increase the uncertainty of interpretation; and database errors in sequence annotations can lead to misunderstandings in the identification. Consequently, we get a huge amount of apparently useless data (for instance, non-matching mass spectra or low-scoring *de novo *interpreted sequences), which most of the times are simply discarded. As a result, this data is seldom accessible to other groups involved in the identification of the same or homologous proteins. Our conviction is that we can benefit from this kind of data making it available as searchable repositories for other laboratories. If we compared data coming from different laboratories then we would be able to eventually discover new matches. The discovery of matches would contribute to further discriminate between really waste data and possibly good data. We envision many advantages with this new methodology, as other laboratories could provide the missing information for an incomplete spectrum or sequence, making a proteine identification process succeed; or even more, matches could help to recognize new proteins or identify PTMs.

### P2P Networks for Proteomics

We propose a new scenario where the information to be searched is no longer centralised in a few repositories, but where information gathered from experiments in peer proteomics laboratories can be searched by fellow researchers. To avoid centralising all data into a single repository --with all the problems that such centralisation would entail--, it is better to maintain the information locally at each of the proteomics laboratories. As a result, this decentralised data storage needs a decentralised search mechanism. The use of peer-to-peer (P2P) technologies fits our needs.

A P2P network provides methods for accessing distributed resources with minimal maintenance cost. It also provides scalable techniques to search through large amounts of resources scattered through the network. Furthermore, joining or leaving the network becomes a simple task. These properties of P2P networks make the technology an ideal candidate to implement a distributed search mechanism in a network of proteomics labs. Other distributed storage systems such as distributed databases or federated storage services have been developed with efficiency in mind, and the maintenance and joining costs for these solutions are very high.

A proteomics laboratory acting as a peer in a P2P network would be able to share its complete or partial data repository --e.g., mass spectra and *de novo *interpreted sequences-- so that other peers can benefit from it. In addition, in order to find matches among data coming from different peers, the interacting peers of such a P2P network would need also to validate and cross check the consistency of the information obtained by fellow peers.

In this article, we describe an approach that implements such a P2P network on top of the OpenKnowledge (OK) system [5,6], which was developed in the scope of the European *OpenKnowledge *project [7].

### The OpenKnowledge System

The OpenKnowledge (OK) system is a fully distributed system that uses P2P technologies in order to share peer-interaction protocols and service components across the network. For this, a kernel module -- the OK kernel-- needs to be installed in each machine that is to be connected to the system. We shall call the protocols and service components to be shared generically *OpenKnowledge Components *(OKCs). Furthermore, these services are executed and coordinated using the same set of tools. In the *Methods *section below we will show how the tools of the OK system are used to implement the proteomics P2P application. The OK system consists of three main services which can be executed by any computer running the OK kernel:

• a discovery service consisting of a distributed hash table (DHT), by which peer-interaction protocols and other OKCs are stored, so that they can be located and downloaded by users;

• a coordination service, which manages the peer interactions between OKCs; and

• an execution service, which is capable of executing the offered service by means of the OK kernel at the local machine.

The workflow for implementing a new application on top of the OK platform is as follows. First, a specification defining the interaction protocol linking different services has to be defined. This specification is published to the discovery service so that other users can find it and can execute OKCs capable of playing the roles specified in the peer-interaction protocol. A developer, not necessarily the one that specified the protocol originally, will develop the OKCs that are to play the roles defined in the protocol specification. Some of these OKCs may be shared across the network by publishing them to the discovery service, so others can also execute them on their local machines. At this point the application is said to be *implemented*.

After the application is implemented, it can be executed on top of the OK system. For this purpose, the users wanting to interact as specified in the given peer-interaction protocol by playing one of the roles will subscribe the appropriate OKCs to it. The discovery service is in charge of managing these subscriptions, and when it gathers enough of these to satisfy all the necessary roles in the protocol, it sends this information to a designated peer acting as the protocol coordinator who will start managing the peer interaction by asking each of the components to provide the services when required by the interaction protocol.

### The Lightweight Coordination Calculus

For the case at hand, the developer has to specify a protocol of the peer interaction defining the roles each perticipating peer has to play, the sort of messages sent amongst them, and the particular constraints to be solved by the OKCs enacting these roles. Several modelling languages such as those reviewed in [8] could have been chosen. Our aim, however, is to use the most easily applied formal language for this engineering task that we could conceive and for which an executable peer-to-peer environment already exists, choosing thus the *Lightweight Coordination Calculus *(LCC) [9].

LCC is the executable interaction modelling language underlying the OK system. It is used to constrain interactions between distributed components and is neutral to the infrastructure used for message passing between components, although for the purposes of this paper we assume components are peers in some form of peer-to-peer network.

**Figure 1:**
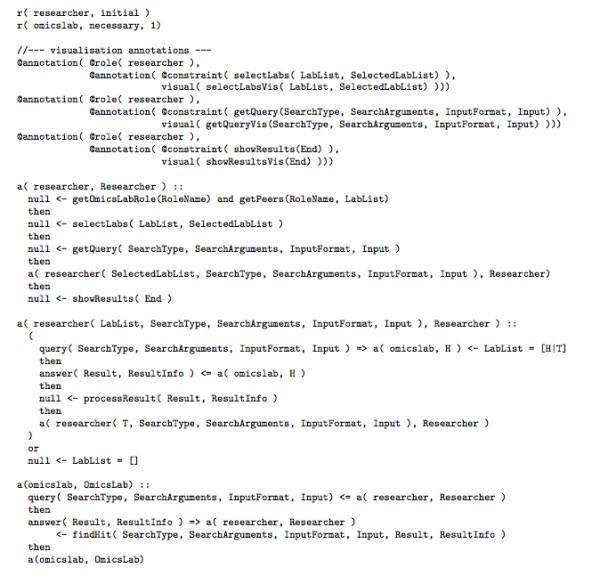
**LCC specification of the protocol for sequenced MS spectra sharing**.

For example, Figure 1 shows the specification in LCC of the protocol for sequenced MS spectra sharing that we will describe in detail later in the *Methods *section. It is based on a simple query-answering protocol between one inquirer and many repliers.

An LCC specification describes (in the style of a process calculus) a protocol for interaction between peers in order to achieve a collaborative task. The nature of this task is described through definitions of roles, with each role being defined as a separate LCC clause. The set of these clauses forms the LCC interaction model. An interaction model provides a context for each message that is sent between peers by describing the current state of the interaction (not of the peer) at the time of message passing. Coordination is achieved between peers by communicating this state along with the appropriate messages. Since roles are independently defined within an interaction model, it is possible to distribute the computation to peers performing roles independently, with synchronisation occurring only through message passing. Should the application demand it, however, LCC can also be used in more centralised, server-based style.

**Figure 2:**
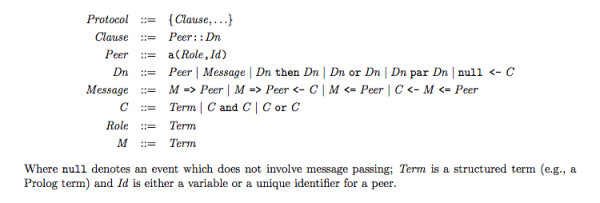
**Syntax of LCC**.

Figure 2 shows the main definitions of LCC's syntax. A detailed discussion of LCC, its semantics, and the mechanisms used to deploy it, lies outside the scope of this paper. For these, the reader is referred to [9]. In this paper, though, we explain enough of LCC to demonstrate how to represent interactions.

An interaction model in LCC is a set of *clauses*, each of which defines how a role in the interaction must be performed. Roles are described in the head of each clause by the *type of role *(and its parameters) and an *identifier *for the individual peer undertaking that role. Clauses may require subroles to be undertaken as part of the completion of a role. The definition of performance of a role is constructed using combinations of the *sequence operator *('then') or *choice operator *('or') to connect messages and changes of role. Messages are *terms*, and are either outgoing to another peer in a given role (' => ') or incoming from another peer in a given role (' <= '). Message input/output or change of role can be governed by a *constraint *to be solved before (when at the right of ' <-') or after (when at the left of ' <-') message passing or role change. Constraints are defined using the normal logical operators for conjunction, disjunction, and negation. If they are subject to fail, the interaction may proceed along alternative paths (e.g., those specified with operator 'or'). Notice that there is no commitment to the system of logic through which constraints are solved --on the contrary we expect different peers to operate different constraint solvers.

A protocol like the one in Figure 1 is generic in the sense that it gives different interactions depending on how the variables (starting with a capital letter) in the clauses are bound at run time --this depending on the choices made by peers when satisfying the constraints within these clauses.

### OpenKnowledge Components

To complete the application, we need also an implementation of the OKCs enacting each of the roles. For the protocol specified in Figure 1, this means two OKCs. One has to enact the *researcher *role as specified in the first two clauses, and another one has to enact the *omicslab *role as specified in the third clause. As a result each OKC will need to be able to solve the constraints occurring in their respective role specification.

For instance, for the *omicslab *role, the relevant OKC must be able to solve the constraint *findHit(...)*. Therefore, its implementation must provide at least a *findHit *method. This method should search the local database for data that matches a given query. Obviously, this implementation will be tightly coupled to the local machinery, the file format used for storing this information, and the type of storage system from where it has to be retrieved. This is an obstacle for the portability of OKCs across different laboratories. Consequently, it is advisable that each laboratory develops its own particular OKC for the *omicslab *role to be played, adjusted to its own system requirements. However, standard OKCs for the most common formats and mass spectrometers could be made publically availabe for dwonload and sharing. There is no restriction in the OK system to prevent locally produced OKCs from being published and downloaded by other users.

## Methods

To show the viability of a P2P-based data-sharing environment for the task of protein identification in proteomics we first specify in LCC a protocol for sharing sequenced MS spectra among peer laboratories. Then we describe the OpenKnowledge components (OKCs) that we have implemented to play the roles specified in the protocol, and finally we recount an actual experiment carried out with the OK system. The aim of the experiment is to serve as *proof of concept *for applying P2P technology to the task of protein and peptide identification. As such, we do not claim that the experiment proves that the OK system for P2P-based data-sharing significantly improves all current standard protein and peptide identification protocols based on centralised database search. The data available for the experiment is insufficient in order to come to such conclusion. However, we do show in the *Results and Discussion *section below that by using a P2P-based data-sharing environment such as the one proposed in this article, researchers gain valuable information that allows them to raise the confidence of their identification task.

For an enhanced selection of those peer laboratories that are to participate in the data-sharing protocol, we have added a confidence evaluation mechanisms that varies over time, and which is based on the expected answer accuracy of peer laboratories.

### Protocol Specification

Figure 1 shows an LCC specification of a protocol that guides peer laboratories in their search of each other's locally stored proteomic data files. This is only one of many possible protocols of this kind. LCC protocols are declarative specifications, and as such they are neutral to the specifics of a protocol execution. The only requirement is that all peers in the network that are to interact by means of a given interaction protocol should be capable of doing so. This capability amounts to (a) running a local copy of the OK kernel, and (b) having a local implementation of an OKC capable of resolving the constraints relevant to the role a peer is playing in the protocol.

For our proof of concept we have specified a protocol --and implemented the required OKCs-- for sharing sequenced MS spectra among peer laboratories. Ideally, to obtain the advantages of peer-based MS spectra sharing as outlined in the *Background *section above, we should ultimately aim at querying and sharing MS spectra directly. However, for our proof of concept for validating the potential gain of peer-based proteomic data sharing, we have first targeted the implementation of a system for *sequenced *MS spectra sharing. Since the protocol models a simple query-answering interaction between one inquirer and many repliers, its application to MS spectra sharing will depend on the availability of OKCs that implement searching based on spectrum-to-spectrum matching. The OKCs we have implemented so far for our proof of concept, allow searching based on sequence-to-sequence matching (by means of BLAST) and spectra-to-sequence matching (by means of OMSSA).

For the actual specification of the protocol, two main roles are needed, one for the inquirer, which in the protocol specification has been termed *researcher *(the clause headed by a(researcher, Researcher)::), and another for the replier called *omicslab*, which will be replying to the queries (the clause headed by a(omicslab, OmicsLab)::). We will start explaining the latter role first, which is simpler.

• *omicslab*: A peer in this role waits for a message with a query from a peer playing the *researcher *role, then solves this query by executing the *findHit *constraint that finds all matching hits in its local database, and finally sends these hits back to the *researcher *peer via another message. This is specified as a conditional message-passing action that is only carried out when the *findHit *constraint can be satisfied.

• *researcher*: A peer in this role acts as the inquirer, and the role makes use of a *researcher *subrole that includes additional parameters. A peer in the main *researcher *role (the one without parameters) asks the user for a query. It does so by launching a input GUI with the constraint *getQuery *and then iterating through all the selected proteomics labs participating in the peer interaction (obtained via constraints *getOmicsLabRole, getPeers *and *selectLabs*). It aggregates all the different results and displays them to the user through an output GUI that is launched by solving the constraint *showResults*. Notice that all these constraints are conditions of an empty message-passing action labelled null. (This is syntactical requirement of LCC: constraints always go together with a message passing action, which can be the empty one.)

The iteration through all the omics laboratories is currently specified to be done via a recursive helper subrole, which receives the query and a list of omics laboratory. This role change is executed after getting all the identifiers of the peers playing the *omicslab *role by means of the *getPeers *constraint, which is executed by the protocol coordinator, who is the peer holding this information. In this subrole, the peer first sends a message containing the query to the first laboratory in the list, then aggregates the response it receives in the message from the laboratory, and finally runs a recursive call to the rest of the list. When the list of laboratories is empty it returns an empty list of results. We could have decided alternatively to specify that queries ought to be sent out in parallel to all selected laboratories. This would be the obvious choice to speed up the querying process, but this is not relevant for the objectives of this article.

Constraints of the *researcher *role that require user input --such as selecting candidate laboratories or writing the query-- and generate output to the user --such as displaying results-- are done via so call *visual constraints*. That is, these constraints are annotated in the LCC specification to be solved by means of domain-specific GUIs. In our case here they are specially tailored for sequenced MS spectra sharing.

### OpenKnowledge Components (OKCs)

In the following we describe the implementation of OKCs that ground the enactment of the protocol of Figure 1. As mentioned above, our implementation so far allows for searching that is based on sequence-to-sequence matching (by means of BLAST) and spectra-to-sequence matching (by means of OMSSA). With this initial implementation we are capable to run our experiment that serves as proof of concept of the proposed P2P proteomics data sharing environment.

#### The researcher OKC

This OKC implements the constraints relevant for a peer that wants to participate in the peer interaction playing the *researcher *role. Hence, the OKC's main task is to ask the user for the proteomic query to solve, forward it to the laboratories, fetch the results, and present them back to the user.

**Figure 3:**
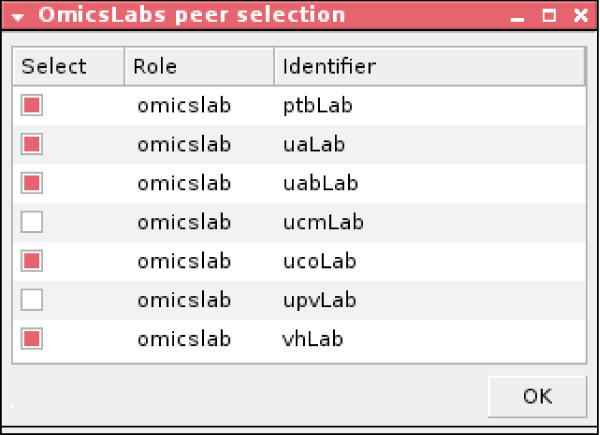
**GUI for selecting peer laboratories for the data-sharing protocol**.

The getOmicsLabRole(RoleName) and getPeers(RoleName, LabList) constraints are used to get the list of omics lab peers participating during a particular enactment of the protocol. The selectLabs(LabList, SelectedLabList) filters those laboratories to which users want to sent the proteomic query. It shows a GUI (Figure 3) by which users identify and select the desired peer laboratories.

The getQuery(SearchType, SearchArguments, InputFormat, Input) constraint asks the user for the proteomic query to solve. It requires four arguments that need to be provided by the user:

• *SearchType*: the type of search to be performed (BLAST or OMSSA).

• *SearchArguments*: the parameters to be used by the laboratories when executing their locally installed search engines.

• *InputFormat*: the proteomic search engines (BLAST and OMSSA) allow different input formats, this argument is used to inform the search engines about the format used in the input.

• *Input*: the proteomic sequences (if BLAST is used) or mass spectra (if OMSSA is used) that constitute the input to the search engines.

**Figure 4:**
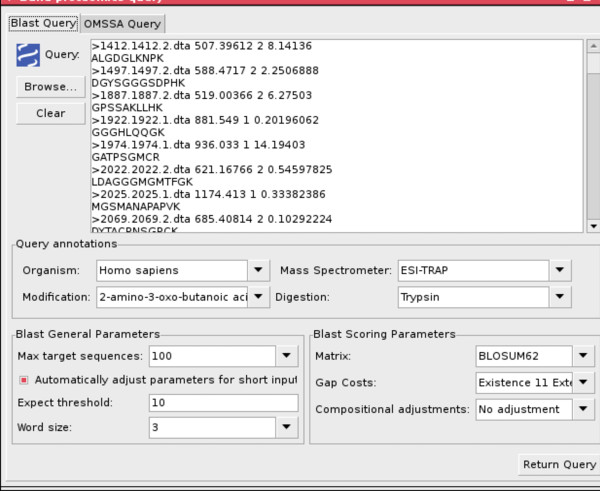
**GUI for building BLAST queries**.

**Figure 5:**
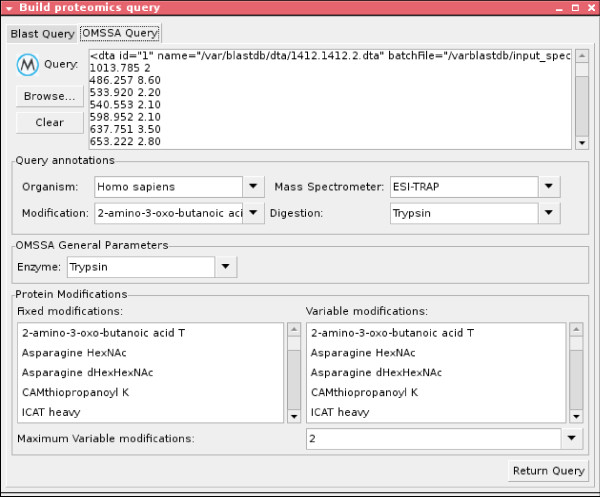
**GUI for building OMSSA queries**.

To solve the getQuery(SearchType, SearchArguments, InputFormat, Input) constraint, a custom visualisation (Figures 4 and 5) is shown to the user. With these GUIs the user can easily build the proteomic query to be submitted to the system by writing or selecting the arguments of the constraint.

As soon as researchers have built their query, it can be sent to each one of the laboratories that are in the filtered laboratory list. This task is done by the researcher(LabList, SearchType, SearchArguments, InputFormat, Input) subrole. This role iterates the list given to *LabList *using recursion; at each iteration the message query(SearchType, SearchArguments, InputFormat, Input) is sent to a laboratory of the list, a(omicslab, H), and the researcher peer waits for the laboratory peer's response message answer(Result, ResultInfo) to aggregate it.

**Figure 6:**
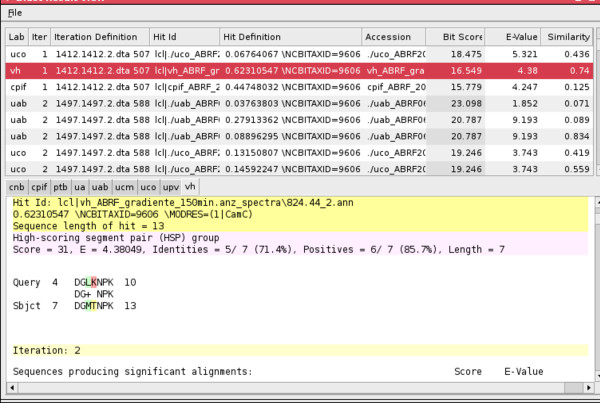
**BLAST result window with answers from labs**.

**Figure 7:**
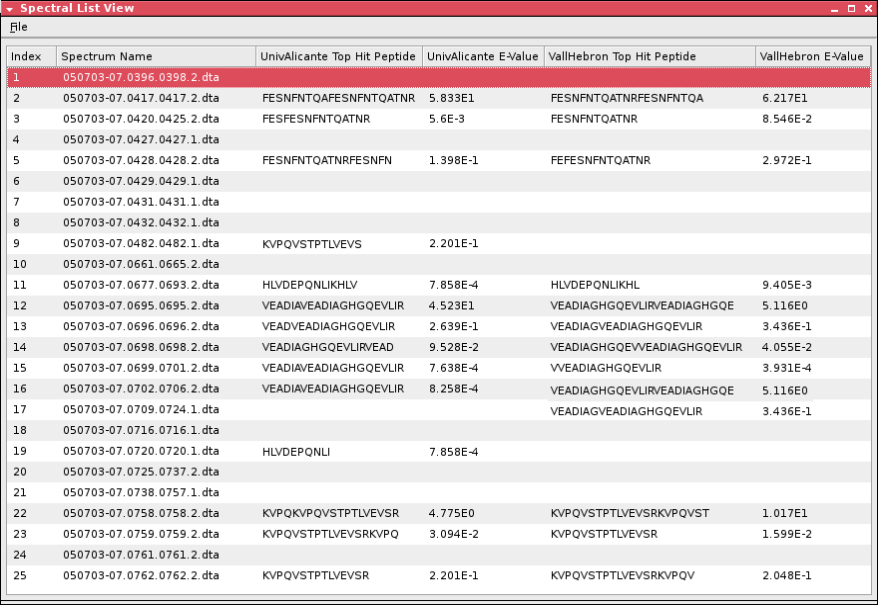
**OMSSA result window with answers from labs**.

**Figure 8:**
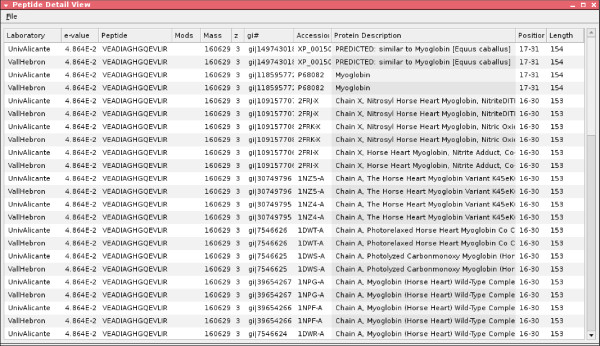
**OMSSA peptide detail view**.

**Figure 9:**
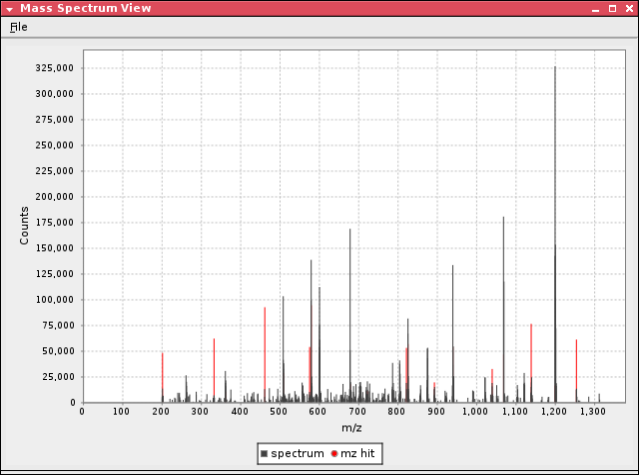
**OMSSA mass spectrum view**.

When all the various results from the laboratories have been collected, the processResults(End) constraint is invoked. This constraint launches another custom visualisation GUI for human users (see Figures 6, 7, 8, and 9), by which they can examine the different results returned by the laboratories.

**Figure 10:**
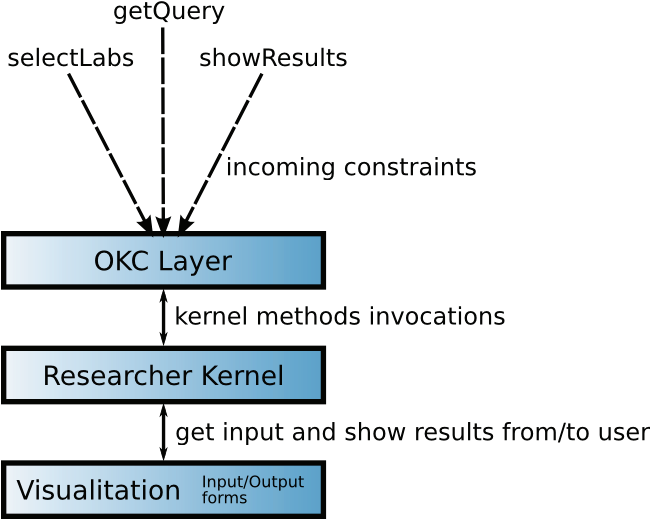
**Schematic view of the researcher OKC architecture**.

From an architectural point of view, the *researcher *OKC has been divided into three main components: the *OKC layer*, the *researcher kernel *and the *visualisation component*. With this division, if the protocol specification or the visualisation requirements are modified, the corresponding changes to the OKC can be applied quickly. The *OKC layer *acts as a thin interface between the OK P2P network and the *researcher kernel*, translating incoming constraints into *researcher kernel *method invocations. The *researcher kernel *contains all the utilities to build the proteomics queries and to parse the results provided by the laboratories, and it invokes the visualisations when needed. A schematic view of the architecture is shown in Figure 10.

#### The omicslab OKC

This OKC implements the only constraint relevant for a peer that participates in the peer interaction playing the *omicslab *role, namely findHit(SearchType, SearchArguments, InputFormat, Input, Result, ResultInfo). This constraint is to solve the query received from a peer in the *researcher *role and to return the result. The *SearchType, SearchArguments, InputFormat *and *Input *arguments are supplied by the *researcher *peer, and they are used as input to solve the query. The *Result *and *ResultInfo *arguments are instantiated by the *omicslab *peer. *Result *contains the result of the execution of the proteomic search engine and *ResultInfo *contains additional metadata about the result.

**Figure 11:**
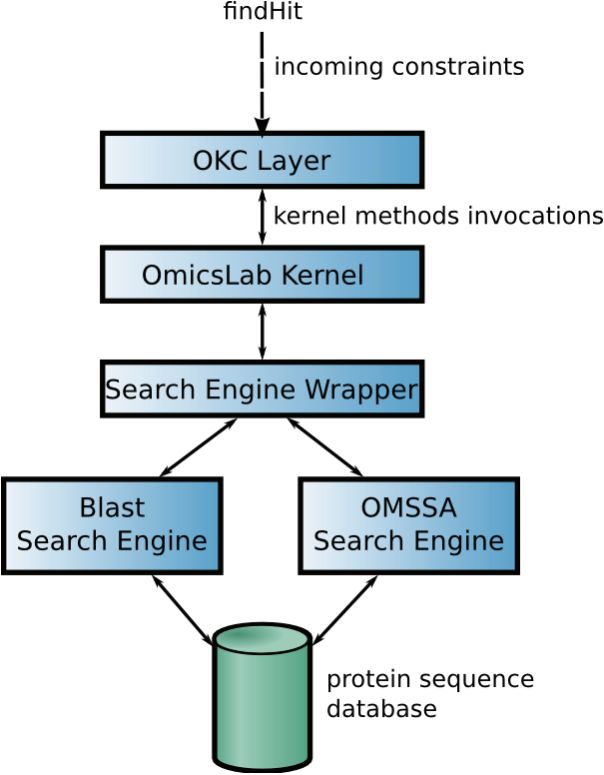
**Schematic view of the omicslab OKC architecture**.

From an architectural point of view, as with the *researcher *OKC, the *omicslab *OKC has been split into three main components: the *OKC layer*, the *omicslab kernel *and the *search engine wrapper component*. The *OKC layer *function is to act as interface between the OK P2P network and the *omicslab kernel*, mapping constraints into *omicslab kernel *functions. The *kernel *task is to identify the incoming query and to send it to the search engines through the *search engine wrapper component*. This latter component executes the locally installed proteomic search engine and returns the result to the *omicslab kernel*. A schematic view of the architecture is shown in Figure 11.

In order to connect a peer playing the *omicslab *role into our OK P2P network it is also necessary to install the BLAST and OMSSA proteomic search engines, to set up the peer's proteomic database, and to configure the peer to use the search engines over its database.

**Figure 12:**
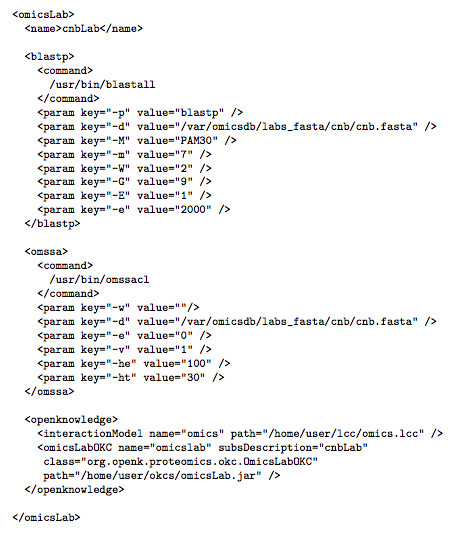
**Fragment of the configuration file used by the omicslab peers**.

• *Search engines: *We decided not to include the search engines as part of the *omicslab *OKC, to make it platform- and search-engine-independent, as BLAST and OMSSA can be freely downloaded from NCBI, the National Center for Biotechnology Information (http://www.ncbi.nlm.nih.gov). It is required to install them locally in every machine acting as an *omicslab*.

• *Databases: *For setting up the protein sequence database with the mass-spectra data returned by the lab's local mass spectrometers we processed each set of *mgf *files (a common format to collect mass-spectra) using the *de novo *interpreter tool PEAKS, which was available to all ProteoRed lab members (see *Experimentation *section below) to obtain a corresponding set of amino-acids sequences. Before building the database of sequences we applied a filter over *de novo *results discarding *short sequences *(less than 4 bases) and duplicates. We assume the first (highest score) sequence to be the best *de novo *interpretation; after that, the *de novo *score is not taken into account anymore, although its value is annotated in the database as header information. The final step consists of formatting these plain text FASTA files to a binary BLAST formatted database (by means of the *formatdb *program provided in the BLAST package). Finally, to also pull existing online proteomic databases into our P2P network we also set up *omicslab *OKCs whose proteomic databases were downloaded from institutions such as NCBI.

• *Configuration: *To configure a peer to use the search engines, each machine acting as *omicslab *peer contains a configuration file. By reading this configuration file the *omicslab *peer knows where the search engines are locally installed, what database it should be using for each search, and the default parameters to use with the search engines. A fragment of a configuration file can be seen in Figure 12.

### Experimentation

For the experimentation we have drawn from real data obtained from the ProteoRed scientific community. In the following we briefly describe this community, the test data employed, how the experimentation has been set up, and the concrete data-sharing peer interaction launched.

#### The ProteoRed Scientific Community

The National Institute for Proteomics, ProteoRed, is a network for the coordination, integration and development of the Spanish proteomics facilities providing services to support Spanish researchers in the field of proteomics. As of 2009 ProteoRed integrated 19 well-established proteomics facilities giving services all over Spain and abroad.

ProteoRed offers major services necessary in all stages of the protein analysis process, and its main objective is to increase the specialisation and competitiveness of proteomics facilities, considering the type of technologies and equipment available, and the type of customers, their expertise and their geographical situation. Customers are research groups from universities, the CSIC, hospitals, or other public institutions, as well as private companies (biotech and pharmaceutical companies). ProteoRed also has the objective of testing new technological developments to provide new proteomics methodologies and equipment to the Spanish proteomics facilities. It also establishes open channels with customers of these proteomics services to know their technological needs, data accuracy, quality requirements, price scales, and new services needed for the future. ProteoRed also takes care of the coordination of courses, workshops and meetings to promote and enhance the quality of proteomics knowledge through the scientific community, ProteoRed technicians and governmental agencies.

#### The Test Data

For our test data we have decided to use preexisting MS/MS data repositories from the 2006 ABRF (Association of Biomolecular Resource Facilities) test sample. It consists of a mixture of 48 purified and recombinant proteins (plus an unknown number of protein contaminants) extensively tested during the ABRF Proteomics Standards Research Group 2006 worldwide survey.

Seventy-eight laboratories participated in the analysis of these mixtures, some of them members of the ProteoRed network. Among these, only 35% could correctly identify more than 40 protein components. Thus, the sample, being relatively handy for the purpose of testing the OK system, is still of enough complexity to become a challenge for most proteomics laboratories.

This sample was prepared by combining five picomole aliquots of each protein. For this purpose, individual proteins were previously purified to assure a purity *>*95%, and the protein concentration determined by amino-acid analysis. The combined sample was lyophilized in 1 mL polypropylene tubes for storage before analysis. The presence of low levels of impurities in the mixture represented an additional challenge to this analysis. Thus, in addition to the 48 standard proteins, most laboratories reported the identification of many other proteins. These identifications could be either due to real contaminants or to false positive identifications. To ascertain which was the case requires a careful analysis of the full data obtained by a laboratory.

This ambiguity could be rapidly solved querying the OK system and searching for other laboratories reporting the same unexpected identifications as we show in the test experiment. This case is an example of a more general situation when a laboratory needs to evaluate the confidence of results that cannot be supported by other means --such as a high confidence match in a database-- by checking if the same data has been obtained by a number of independent partners working with similar biological samples.

#### Experiment Setup

To test whether the OK system could be used by the ProteoRed community in order to speed up the protein detection process, we simulated an environment in which several peers of the OK system were emulating real proteomics laboratories. Through this environment a researcher could query these peer laboratories to retrieve data from their local databases.

Since we did not have access to the entire MS/MS repository of the 2006 ABRF test sample, we set up a P2P ntework of those 9 laboratories of the ProteoRed community that made their data available for our experimentation. Each of these peers managed its own database containing protein data extracted from the ABRF test sample. To serve as an interface between the ABRF database and the OK system we implemented an *omicslab *OKC for each peer and subscribed it to play the *omicslab *role in charge of replying to incoming proteomic queries as specified in the MS spectra-sharing protocol of Figure 1. In addition, we gave proteomics researchers a tool that allowed them to search for proteomic information through the OK P2P network by sending queries to *omicslab *peers and retrieving data from their databases. For this, researchers had to set up their access to the OK system by executing the following set of steps:

1. *Installing the OK kernel*. All researchers need to link into the OK system by installing the OK kernel [6] on a computer with an internet connection, in any operating system, with the only requirement that it has the Java 1.5 suite installed.

2. *Searching for the protocol specification*. The OK system supports that different peers interact according to peer-interaction protocols. These protocols are to be specified and made public in the OK system. Users of the OK system can then search for protocol specifications that define the type of interaction according to which they would like to interact with other peers. In our current scenario researchers will use the browsing facility provided by the user interface of the OK kernel application to search for the appropriate protocol specifications. Searching is achieved by sending queries with keywords to a discovery service, which is in charge of storing all of the published OKCs and protocols. (The discovery service is itself not a centralised, but a completely distributed service that follows the decentralised approach taken by P2P networks.) The discovery service then retrieves all the protocol specifications whose metadata matches the query. The researchers can then read the descriptions associated to the retrieved protocol specifications in order to select the one that suits them best. If no specification suits them, they can refine the query by using different keywords.

3. *Installing the researcher OKC*. Recall that the protocol specified for our experiment defines two roles: the *omicslab*, which is in charge of replying to queries, and the *researcher*, which is the one sending out queries (see the *Protocol Specification *section above). Actual researchers that want to query peer laboratories will have to do so by enacting the *researcher *role as specified in the protocol. For this they must have previously installed an OKC capable of solving the constraints attached to that role (see the *OpenKnowledge Components (OKCs) *section above). OKCs can also be published in the OK system so that it is easy for users to find them and install them locally on their computers. Downloading and installing an OKC from the OK system can be achieved directly from the kernel's user interface. Once both the protocol specification and the role that a user wants to play have been chosen, one needs to search for existing OKC implementations for the given role, and then download and install them. At this point a researcher would be ready to start launching proteomic queries. Although this is the simplest way to install the required OKC, an advanced user may also find or develop an OKC through other means and plug it into the kernel.

4. *Subscribing to the researcher role*. Once the actual researchers have installed the OKC needed for playing the *researcher *role, they can start the peer interaction through a subscription. Users select the protocol specification and role they want to play and then run the subscription command from the user interface. This command sends the subscription information to the discovery service, which will define a peer (the researcher or another peer in the network) that will act as a coordinator of the peer interaction, following the protocol that will govern the peer interaction between the researcher and those peers that have subscribed to play the other roles defined in the protocol. In our case these will be the laboratory peers subscribed to play the *omicslab *role.

#### Protocol Enactment

When the protocol and OKCs are in place, and sufficient peers have subscribed to the required roles of the protocol, the protocol itself can be enacted.

1. *Selecting the laboratories*. When the interaction starts, the protocol iniciator peer recevies the list of all the peers that have subscribed to the *omicslab *role is received by the peer. This list is shown to the user which has to select the subset of the labs that he or she wants to query.

2. *Building the proteomic query*. Having selected the laboratories to which the query will be sent, the user will then have to create the query. This is also done through a user interface providing users with a form through which they can specify the query. This form consists of:

• an item asking to select the type of search (BLAST or OMSSA);

• a text box in which to write the proteomic text query or import it from a file;

• another text box where the researcher can add meta-data annotations that are used if the confidence on the returned results is to be determined (see the next subsection); and

• a subform where the user can enter custom search arguments to be used by the search engines.

Once the query has been introduced by the researcher it is sent to all the selected *omicslab *peers so that they can process it and reply with the set of matching proteomic data.

3. *Showing the results from laboratories*. Every time an *omicslab *peer replies to the query, the *researcher *OKC stores the results. Once all the *omicslab *peers to which the query was sent have replied, the results are shown to the users via a custom visualisation. Through this custom GUI researchers can browse through the different results and compare them. This is the final step of the protocol execution, if the researchers want to make another query they simply start another interaction.

### Confidence in Peers

In our scenario, researchers send queries to and receive answers from a set of peer proteomics laboratories. For these researchers it is important to have a mechanism helping them to distinguish which laboratories return more significant and relevant answers to their queries. This can be achieved by measuring the confidence that researchers have in each laboratory, a confidence that is built during successive queries launched by the researcher to their peer laboratories.

In [10], the trust on a peer (a proteomics lab in our scenario) is defined as the overall satisfaction with previous experiences with that peer. In our case the satisfaction measure of each particular experience with a laboratory is based on the similarity between the query and the answer obtained from the laboratory. This similarity, however, is not only the similarity between amino-acid sequences -- it also takes into account additional information such as the enzyme chosen for digestion during sample preparation, the type of mass spectrometer used, or the kind of organism that the sample was taken from, among other information.

The similarity between a query and a laboratory hit (the basic building block of the confidence calculation in this application domain) is then defined as the product of two factors:

spectrum similarity = spectrum significance ×protocol similarity

where the value of *spectrum significance *represents how significant the matched spectrum is with respect to the query spectrum, and the value of *protocol similarity *represents how similar the spectrographic protocols are that where followed by the researcher when obtaining the spectrum in the query and the laboratory when obtaining the spectra in its database. Let us explain these two similarity measures in more detail.

#### Spectrum significance

To calculate the significance of a spectrum (or of its associated sequence of amino-acid characters) search engines that work over databases of amino-acid sequences usually report a score *S *together with a probabilistic value, referred to as *P*-value. The score *S *is a measure of the similarity of the query to the sequence matched, and the *P*-value is a measure of the reliability of this score. It is the probability due to chance that there is at least another match with score greater than or equal to *S*. (Here *chance *means the comparison of (i) real but non-homologous sequences; (ii) real sequences that are shuffled to preserve compositional properties; or (iii) sequences that are generated randomly based upon a DNA or protein sequence model [11].) But instead of determining *P*-values directly, search engines such as BLAST or OMSSA report the so-called *E*-values, which are easier to interpret. The *E*-value is the number of times a sequence with a score greater than *S *may occur by chance in the database. That is, the closer the *E*-value to zero, the better the match. But as *E*-values are not normalised in 0[1], we take the *P*-value for computing spectrum significance, which can be derived from the reported *E*-value as follows: *P *= 1 - *e*^-*E*^.

The second factor that we consider is the number of significant spectra in the set *M *of matched sequences reported by a peer laboratory with respect to the score distribution of the sequences of *M*. Thus, given a query sequence, the overall significance of the set of matched sequences provided by a peer laboratory is given by $\frac{\sum_{n\in N}S_{n}}{|N|}$, where *N *= {*m *∈ *M *| *S_m _> σ_M _*}, being *σ_M _*the standard deviation of scores in the population *M*.

#### Protocol Similarity

In its graphical representation a mass spectrum is a plot of the mass to charge values of the detected ions versus their corresponding intensity. Fragmentation spectra from peptides show profiles that are characteristic of the peptide sequence and that contain different types of ions [12]. In addition to the returned spectra, researchers need to have some confidence that the way in which the spectrum in the query has been obtained is comparable to the way in which the hits are obtained. This is because, although the protocol followed by a laboratory may be well defined, the protocol itself admits certain variations that will produce spectra with different ion types. (Bear in mind that this is not the peer-interaction protocol specified in LCC for data sharing in our P2P network.) These variations include the enzymes used to modify and to digest the amino-acids, and the type of mass spectrometer used to produce the spectra.

Another important factor is the organism from which the protein has been obtained. All this information is provided as metadata. We define the protocol similarity of a hit between a query and a database entry as

$$\begin{array}{c} \text{protocol similarity }=\text{ organism similarity }\times \text{ modification similarity }\times \text{ digestion similarity}\\ \;\;\;\;\;\;\;\;\;\times \;\text{mass - spectrometer similarity} \end{array}$$

##### Organism similarity

**Figure 13:**
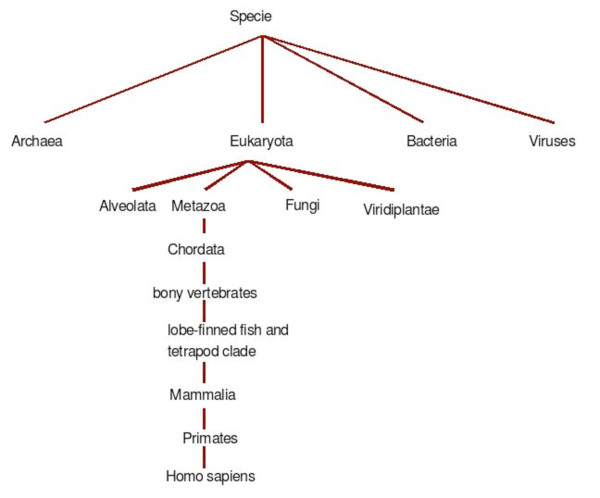
**Fragment of the organism lineage tree**.

The semantic similarity among organisms *o*_1 _and *o*_2 _used for our confidence evaluation is based on the organism taxonomy tree as according to the NCBI lineage. Figure 13 shows a fragment of this taxonomy.

To defined a similarity measure we have used the one described in [13] and repeated here:

$$Sim\left(o_{1},o_{2}\right)=\left\{\begin{array}{cc} 1 & \text{if}\;o_{\text{1}}\text{ = }\;o_{\text{2}}\\ e^{-\kappa_{1}l}\cdot \frac{e^{\kappa_{2}h}-e^{-\kappa_{2}h}}{e^{\kappa_{2}h}+e^{-\kappa_{2}h}} & \text{otherwise} \end{array}\right)$$

where *l *is the length (i.e., number of edges) of the shortest path between nodes, *h *is the depth of the deepest node subsuming both nodes, and *κ*_1 _and *κ*_2 _are parameters balancing the contribution of shortest path length and depth respectively. For example, the path to humans is:

cellular organisms; Eukaryota; Fungi/Metazoa group; Metazoa; Eumetazoa; Bilateria; Coelomata; Deuterostomia; Chordata; Craniata; Vertebrata; Gnathostomata; Teleostomi; Eu-teleostomi; Sarcopterygii; Tetrapoda; Amniota; Mammalia; Theria; Eutheria; Euarchontoglires; Primates; Haplorrhini; Simiiformes; Catarrhini; Hominoidea; Hominidae; Homo/Pan/Gorilla group; Homo; Homo sapiens

and the paths to the rat, the sole, and the rhesus macaque are, respectively:

• cellular organisms; Eukaryota; Fungi/Metazoa group; Metazoa; Eumetazoa; Bilateria; Coelomata; Deuterostomia; Chordata; Craniata; Vertebrata; Gnathostomata; Teleostomi; Euteleostomi; Sarcopterygii; Tetrapoda; Amniota; Mammalia; Theria; Eutheria; Euarchontoglires; Glires; Rodentia; Sciurognathi; Muroidea; Muridae; Murinae; Rattus

• cellular organisms; Eukaryota; Fungi/Metazoa group; Metazoa; Eumetazoa; Bilateria; Coelomata; Deuterostomia; Chordata; Craniata; Vertebrata; Gnathostomata; Teleostomi; Euteleostomi; Actinopterygii; Actinopteri; Neopterygii; Teleostei; Elopocephala; Clupeocephala; Euteleostei; Neognathi; Neoteleostei; Eurypterygii; Ctenosquamata; Acanthomorpha; Euacanthomorpha; Holacanthopterygii; Acanthopterygii; Euacanthopterygii; Percomorpha; Pleuronectiformes; Soleoidei; Soleidae; Solea; Solea senegalensis

• cellular organisms; Eukaryota; Fungi/Metazoa group; Metazoa; Eumetazoa; Bilateria; Coelomata; Deuterostomia; Chordata; Craniata; Vertebrata; Gnathostomata; Teleostomi; Euteleostomi; Sarcopterygii; Tetrapoda; Amniota; Mammalia; Theria; Eutheria; Euarchontoglires; Primates; Haplorrhini; Simiiformes; Catarrhini; Cercopithecoidea; Cercopithecidae; Cercopithecinae; Macaca; Macaca mulatta

Taking as parameter values *κ*_1 _= 0.02 and *κ*_2 _= 0.6 we get the following similarities:

$$\begin{array}{ccc} Sim\text{(homo sapiens; solea) } & \text{ = 0}\text{.46} & \\ Sim\text{(homo sapiens; rattus) } & \text{ = 0}\text{.72}\\ Sim\text{(homo sapiens; macaca mulatta) } & \text{ = 0}\text{.81} \end{array}$$

The NCBI database of organisms and taxonomies is dynamic and very large (currently there are more that 300,000 organisms), but it provides a REST web service to get taxonomic information without the need to download the entire database. (REST is a method to make web service queries where the query is written as an URL over HTTP.)

##### Modification similarity

To calculate the similarity of modification terms, rather than a tree distance we have used a binary similarity table (see Table 1). This is because there are situations that cannot be compared.

**Table 1:** Similarity table for peptide modification

modification code	-1	1	2	3	5	31	32	89	90
-1	1								
1	1	1							
2	1	0	1						
3	1	0	1	1					
5	1	0	1	1	1				
31	1	0	1	1	1	1			
32	1	0	1	1	1	1	1		
89	1	1	0	0	0	0	0	1	
90	1	1	0	0	0	0	0	0	1

-1 = nothing; 1 = oxidation of M; 2 = carboxymethyl C; 3 = carbanidomethyl C; 5 = propionamide C; 31 = carbamylation of K; 32 = carbamylation of n-trem peptide; 89 = oxidation of H; 90 = oxidation of W. For the remaining codes the similarity is 1 if they are equal and 0 if the are not equal.

For example, a peptide modified with oxidation of an amino-acid cannot be compared with another peptide that has not been modified at all.

##### Digestion similarity

To calculate the similarity of the digestion terms we assign a similarity value of 1 between identical enzymes, and also between Trypsin and LysC, but only if the peptide ends with K. In all other cases we assign a similarity value of 0.

##### Mass-spectrometer similarity

**Figure 14:**
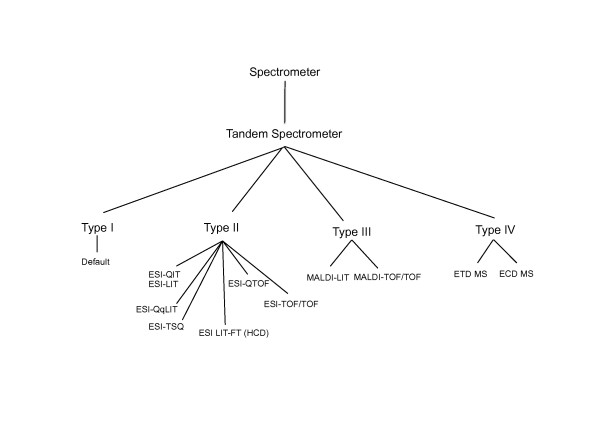
**Similarity tree for different mass spectrometers**.

Finally, the similarity of the mass spectrometers is calculated based on the taxonomy tree depicted in Figure 14 (see Table 2 for acronyms). The tree classifies the spectrometers according to the type of ion fragmentation profiles that are typically produced. The main classification parameters are the ionization method (MALDI or ESI ionization), which determines the type of precursor ions formed, and the collision energy (high- or low-energy collision).

**Table 2:** Mass spectrometer acronyms

acronym	mass spectometer
ESI	electrospray
MALDI	matrix assisted laser desorption
QIT	quadrupole ion trap
QqLIT	hybrid quadrupole-linear ion trap
TSQ	triple stage quadrupole
QTOF	hybrid quadrupole-time of flight
TOF	time of flight
LIT-FT(HCD)	hybrid LIT-Orbitrap with high collision dissociation
ETD	fragmentation by electron-transfer dissociation
ECD	fragmetnation by electron-capture dissociation

## Results and Discussion

To play the *researcher *role we randomly selected 38 peptide sequences obtained by PEAKS *de novo *analysis of the mass spectrometric analytical data obtained by the LP CSIC/UAB proteomics laboratory from the ABRF sample. Additionally we included data derived from spectra that during the original analysis matched to proteins not included in the standard ABRF list.

The mass spectrometric data set from LP CSIC/UAB was obtained by LC-MS/MS analysis of the tryptic digest of the protein mixture in the ABRF sample. This data set included 2000 spectra from which 48 of the 49 proteins in the ABRF standard could be identified by conventional proteomic data analysis. Each protein was identified from the sequence of one or more of its tryptic peptides. Queries were performed against 9 databases, including 7 proteomics labs, the NCBI Swiss-Prot database, and the database of the researcher itself. Making the researcher laboratory play both roles in the peer interaction (*researcher *and *omicslab*) served as a true positive control to check for failures in the functionality our system.

To evaluate the evolution of the confidence of reported answers to queries, the 48 sequences were divided into 4 groups to be able to mimic the building of a query history. Despite that this model does not produce a valid history (as sequences were grouped randomly), it will allow to evaluate the functionality of the confidence calculation.

**Figure 15:**
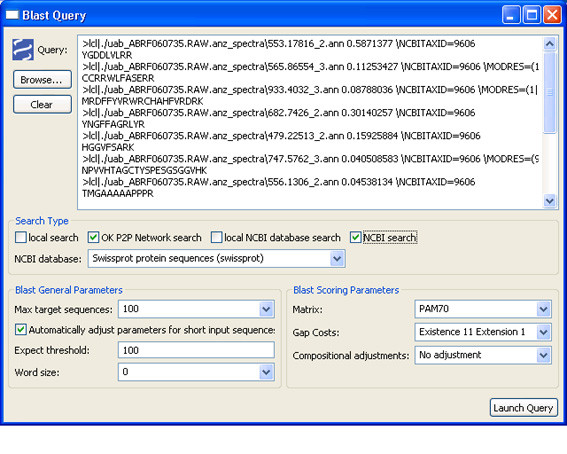
**Query window and BLAST search parameters used for this study**. The sequences shown in the image correspond to the first group of queries.

**Figure 16:**
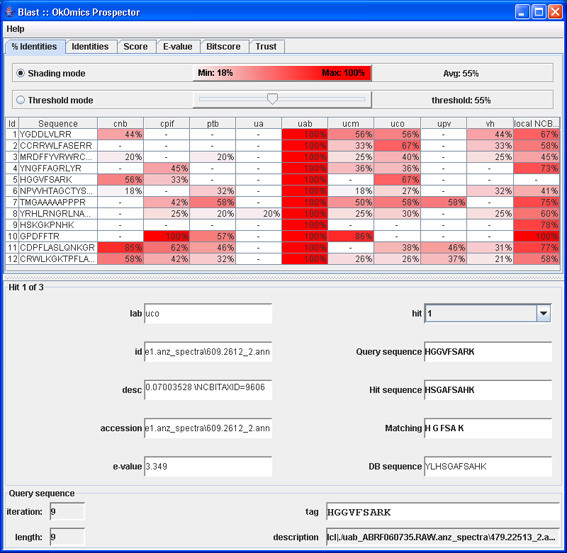
**OK-omics Prospector window**. Responses to the first group of queries (% coincidences).

**Figure 17:**
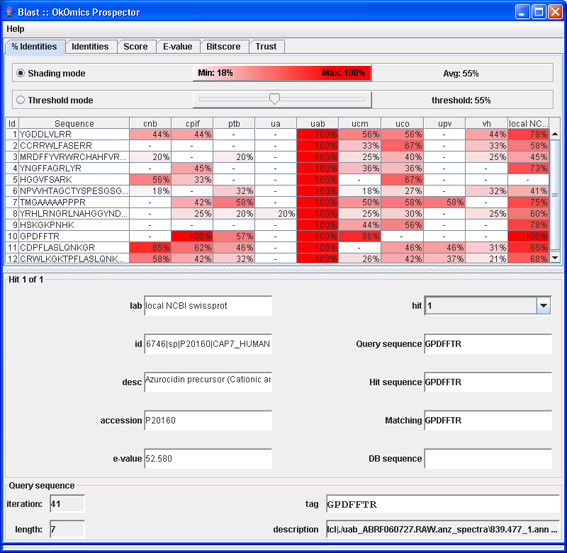
**Match data from ncbi database for query 10**.

Each group was queried with the same parameters (Figure 15) and the results analysed in the researcher OKC prospector window (Figure 16). As expected, the search in the researchers database (column labelled with *uab*) generated always full coincidences. Contrarily, other proteomics labs and the NCBI Swiss-Prot database (labelled with *ncbi*) produced more diverse results. Most of the queries produced high percentage identity values in the *ncbi *search. These hits give direct information about the identity of the peptide and the source protein ('id' and'des' text windows in Figure 17). One of the queries in Figure 17 (Query 10) produced a 100% coincidence in the NCBI Swiss-Prot database. The expectation values for this match indicated that it was not due to hazard. The protein that had been tentatively identified, P20160 (azurocidin precursor) was, however, not included in the list of component of the standard ABRF sample.

**Figure 18:**
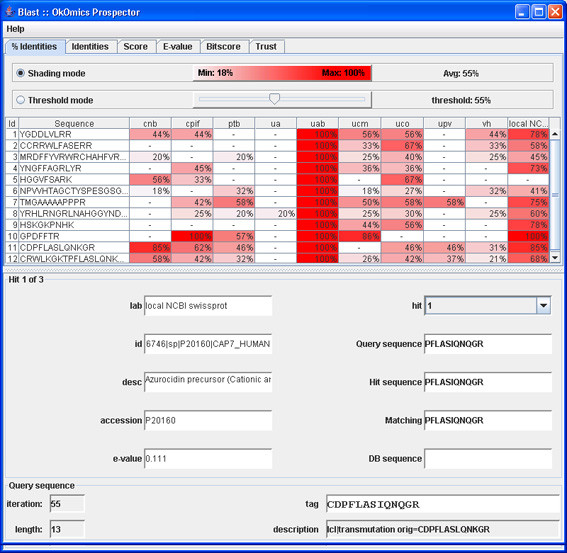
**Match data from ncbi database for query 11**.

The analysis of the answers from other proteomics labs for this sequence showed that other laboratories found identical (see, i.e., *cpif) *or highly homologous sequences (see, i.e., *ucm*). This fact indicated that several laboratories had observed the presence of the same component in their samples and supported the fact that the queried sequence was not the result of noise or lab-specific sample preparation artifacts. More detailed analysis of the results indicated that the presence of protein P20160 was supported by other NCBI matches (see, i.e., Query 11 in Figure 18) and that the corresponding query also had produced highly scored matches in many proteomics labs. This analysis clearly showed that P20160 was a relatively high concentrated contaminant (several laboratories detected several of its tryptic peptides) that could have been present in the ABRF sample.

This fact is evident from the information that can be derived from the OK system. However, it is not straightforward to arrive to the same conclusion by conventional means, as it is difficult to discard organic contamination of the samples. The actual presence of this artifact was only stated after the ABRF study of all the identification reported by the laboratories.

**Figure 19:**
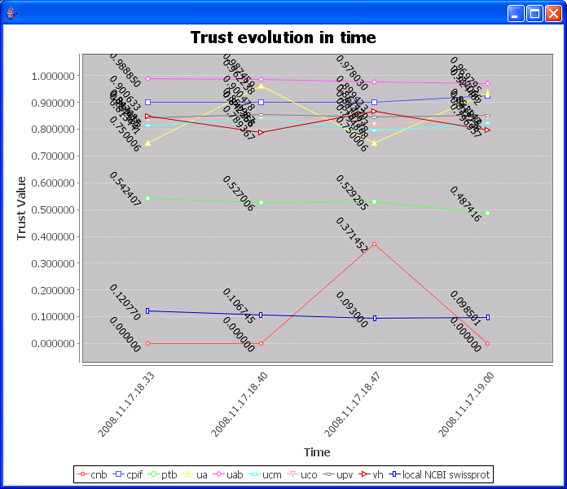
**Confidence evaluation**.

The confidence on the result of each proteomics lab was evaluated for the 4 queries performed (Figure 19). Confidence values for the different laboratories are in the range from near 0 to near 1 indicating different efficiencies sending back high score matches for the queried sequences. No improvements on the quality of the information derived from the OK-omics system were observed by selecting 2-3 of the more trusted labs for these queries. Due to the small size of the databases, an important fraction of the processing time was due to the public NCBI database search. Selecting a few laboratories of high trust could however increase the performance when a higher number of peers are involved in the interaction. As expected by the origin of the data (sequences randomly taken from the LP CSIC/UAB data set) trust values are stable over the experiments.

## Conclusions

We have presented a new form of data sharing for expression proteomics with the aim of (1) augmenting significantly the percentage of peptides and proteins to be sequenced and identified by means of mass-spectrometry-based analysis, and (2) reducing significantly the sequencing and identification time needed. For this we have combined current bioinformatics techniques for proteomics with a novel multiagent system architecture and a distributed knowledge coordination mechanism in peer-to-peer networks, which have been developed in the context of the OpenKnowledge EU project.

In this article we have specified the data-sharing peer-interaction protocol for P2P proteomics, implemented the P2P data-sharing system using the OpenKnowledge system, and carried out a feasibility experiment with test data from preexisting MS/MS data repositories from the 2006 ABRF test sample provided by different laboratories for the ProteoRed scientific community.

We conclude that by using the proposed P2P data-sharing system and protocol a researcher is capable of deriving information from the test data that is not straightforward to obtain by conventional means. This in turn shows that P2P data-sharing in proteomics can indeed lead to enhanced protein identification.

## Competing interests

The authors declare that they have no competing interests.

## Authors' contributions

MS, JA, and CS conceived the P2P-based spectra-sharing method and experimentation, and they specified the peer-interaction protocol. DC and LB implemented OKCs and GUIs, and made the computational setup of the experiment, which was validated and interpreted by JA. CS and EJ designed the confidence evaluation module, which EJ further implemented and tested. AP and DC adjusted the OK system to the spectra-sharing platform. MS wrote the initial versions of the article based on contributions of AP, MA, LB, DC and EJ; and prepared the final version. All authors read and approved the final manuscript.
